# Pulmonary Rehabilitation: The Reference Therapy for Undernourished Patients with Chronic Obstructive Pulmonary Disease

**DOI:** 10.1155/2014/248420

**Published:** 2014-02-19

**Authors:** Nikolaos Samaras, Dimitrios Samaras, Arnaud Chambellan, Claude Pichard, Ronan Thibault

**Affiliations:** ^1^Department of Internal Medicine, Rehabilitation and Geriatrics, Geneva University Hospital, Rue Gabrielle-Perret-Gentil 4, 1211 Geneva 14, Switzerland; ^2^Nutrition Unit, Geneva University Hospital, Rue Gabrielle-Perret-Gentil 4, 1211 Geneva 14, Switzerland; ^3^Explorations Fonctionnelles, Hôpital G et R Laennec, Boulevard Jacques Monod, 44093 Nantes Cedex 1, France

## Abstract

Chronic obstructive pulmonary disease (COPD) combines the deleterious effects of chronic hypoxia, chronic inflammation, insulin-resistance, increased energy expenditure, muscle wasting, and exercise deconditioning. As for other chronic disorders, loss of fat-free mass decreased survival. The preservation of muscle mass and function, through the protection of the mitochondrial oxidative metabolism, is an important challenge in the management of COPD patients. As the prevalence of the disease is increasing and the medical advances make COPD patients live longer, the prevalence of COPD-associated nutritional disorders is expected to increase in future decades. Androgenopenia is observed in 40% of COPD patients. Due to the stimulating effects of androgens on muscle anabolism, androgenopenia favors loss of muscle mass. Studies have shown that androgen substitution could improve muscle mass in COPD patients, but alone, was insufficient to improve lung function. Two multicentric randomized clinical trials have shown that the association of androgen therapy with physical exercise and oral nutritional supplements containing omega-3 polyinsaturated fatty acids, during at least three months, is associated with an improved clinical outcome and survival. These approaches are optimized in the field of pulmonary rehabilitation which is the reference therapy of COPD-associated undernutrition.

## 1. Introduction 

**Figure 1:**
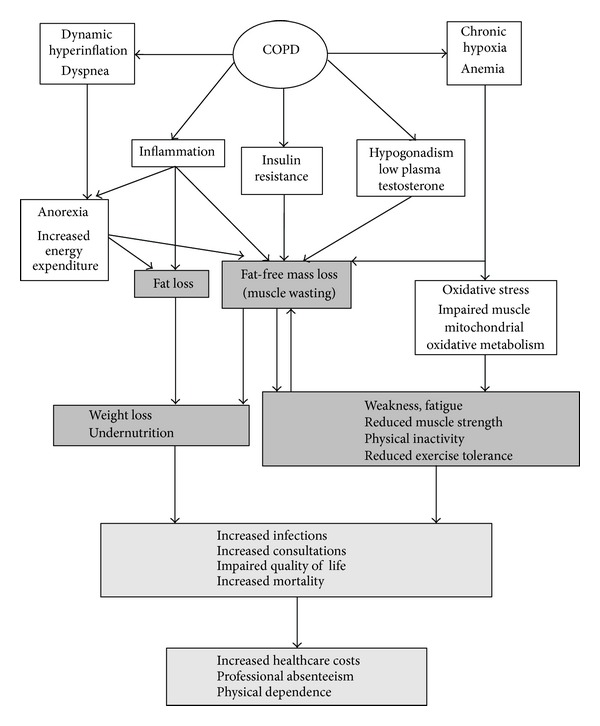
Mechanisms and clinical consequences of undernutrition in COPD patients (from [6]). The white boxes represent the metabolic mechanisms or features, and the grey boxes represent their nutritional clinical consequences. Dyspnoea is mainly attributable to dynamic hyperinflation [2]. Dynamic hyperinflation is the consequence of the loss of lung elastic recoil and increase in airway resistance, thus leading to air trapping, ventilation inefficiency, and increase in work of breathing through increase in inspiratory muscle load [28]. Dyspnoea and inflammation increase in energy expenditure and induce decreased food intake and anorexia. Other features of COPD are insulin resistance, low plasma testosterone, and chronic hypoxia associated with anemia. Altogether these conditions induce undernutrition, characterized by muscle wasting and fat loss. Chronic hypoxia and anemia are responsible for oxidative stress and impaired muscle mitochondrial oxidative metabolism. These results in weakness, fatigue, reduced muscle strength, physical inactivity, and reduced exercise tolerance, which are all favored by muscle wasting. The consequences of undernutrition are increased risk of infections, number of medical consultations, impaired quality of life, and worse survival. This worse clinical outcome has economic consequences: increases in healthcare costs, professional absenteeism, and physical dependence.

The natural history of COPD is poorly understood. It is commonly assumed that chronic bronchial inflammation and small airway narrowing secondary to tobacco smoke take place for many years before any obstructive ventilatory defect could be detected [1]. The main and most serious symptom reported by COPD is dyspnea as a consequence of the mechanical constraints of the obstructive ventilatory defect, which increase during exercise leading to physical inactivity and handicap [2–4]. The combined effects of systemic inflammation and tissue hypoxia partially explain several comorbidities often associated with COPD at a higher frequency than in non-COPD smokers: cardiovascular diseases, osteoporosis, diabetes, and metabolic syndrome [5]. COPD shares similar characteristics with other chronic organ insufficiencies, chronic infections, and cancer: anorexia, inflammation, insulin-resistance, hypogonadism, and anemia [6]. Moreover, increased resting energy expenditure has been reported in COPD patients [7]. These conditions are responsible for fat loss and muscle wasting [8], leading to weight loss [9], muscle weakness, and fatigue (Figure 1). COPD-related weight loss is affecting mostly muscle mass [10, 11] and may be found in almost 40–50% of patients at severe stages of the disease [8]. As muscle wasting is affecting skeletal as well as respiratory muscles, COPD-associated chronic undernutrition and muscle wasting lead to physical inactivity [4, 12], exercise deconditioning [13], and impairment of respiratory function [14], resulting in impaired quality of life [4, 12], increase in bronchial infections, general practitioner consultations [15], and reduced survival [4, 12, 16–26]. This, in turn, increases COPD-related costs (Figure 1). As the prevalence of COPD is expected to increase in future decades [27], the medicoeconomic burden of COPD-associated undernutrition will increase. The impact of undernutrition and fat-free mass loss on clinical outcome and survival is independent of respiratory parameters [16–19]. Several studies have shown that fat-free mass loss measured by bioimpedance analysis (BIA) or midthigh CT scan is a stronger predictor of mortality than body mass index [16, 20, 21, 24]. Therapies aiming at specifically managing COPD-associated nutritional disorders and improving muscle mass are mandatory to reduce the negative impact of the disease on physical activity, quality of life, and clinical outcome.

## 2. Physiopathology of Muscle Wasting in COPD 

As renal or chronic heart failure, the musculoskeletal system is profoundly altered in COPD patients. Muscle is characterized by decreased capillary density and a shift from the aerobic to the anaerobic glycolytic pathways: decreased diameter and smaller fraction of type I muscular fibers [29, 30] together with an increase in type II nonoxidative muscular fibers [31, 32] and decreased expression of aerobic glycolysis enzymes [33, 34]. These alterations are mainly related to tissue hypoxia and inflammation. Both provoke oxidative stress and stimulate the activity of the ubiquitin-proteasome pathways responsible for protein breakdown [35–38], and, in turn, muscle wasting, physical inactivity, and sedentarity. Increased protein catabolism is observed during COPD acute exacerbations [39] and during the whole course of COPD due to systemic inflammatory reaction. Protein catabolism is driven by the secretion of proinflammatory cytokines: tumor necrosis factor-*α* (TNF-*α*), interleukin-1 (IL-1), interleukin-6 (IL-6), interleukin-8 (IL-8), and interferon-*γ* (INF-*γ*). The conservation of an oxidative muscle phenotype could protect myofibers from pathological insults, as shown in mice with chronic heart failure [29, 40]. Therefore the preservation of muscle mass and function, through the protection of the mitochondrial oxidative metabolism, is an important challenge in the management of COPD patients. Recent evidence suggests that pulmonary rehabilitation including physical exercise, oral nutritional supplements, and androgen therapy could improve muscle mass and function and the outcome of COPD patients [41].

## 3. Evidence for Androgen Therapy in COPD

In COPD patients, plasma concentrations of anabolic hormones, such as growth hormone (GH) and testosterone, are frequently decreased [38, 42–45]. A prevalence of low plasma testosterone, that is, hypogonadism, of 38% was reported considering a cut-off of free plasma testosterone of 50 pg/mL in a group of men with severe COPD and a mean age of 70 years [43]. Others have reported a prevalence of 22% [44] and 69% [45], which is higher than age-related hypogonadism. Indeed, total testosterone levels less than 320 ng/dL were found in 17% of patients aged between 40 and 79 years [46], and free testosterone levels less than 6.5 ng/dL were found in 32% of patients aged between 73 and 78 years [47]. In contrast to age-related hypogonadism, lower testosterone plasma concentrations in COPD are mostly associated with a decrease in luteinizing hormone (LH) and follicle stimulating hormone (FSH) hypophysis secretion [43, 48]. COPD severity and hypoxia are related to lower plasma concentrations of LH and FSH [49]. In COPD patients, one study showed an improvement in testosterone plasma concentrations after one month of oxygen therapy [50]. Chronic systemic corticosteroid treatment may alter pituitary stimulation by gonadotropin releasing hormone (GnRh) and thus decrease LH and testosterone synthesis and secretion [45]. Moreover, chronic inflammation, through the effects of TNF-*α* on the hypothalamus-hypophysis-gonadal axis, may participate in reducing testosterone plasma concentrations in COPD [50]. In the general population, low plasma concentrations of testosterone have been related to a decreased muscular mass [51–53] and force [51, 54, 55]. Quadriceps muscle weakness is related to low circulating levels of testosterone in men with COPD [56]. Testosterone increases muscle protein synthesis [57, 58], reduces adipocyte, and increases muscle cells proliferation [57]. Testosterone also inhibits leptin and stimulates ghrelin production which in its turn stimulates GH secretion [11, 57] and appetite. In addition to hypogonadism, reduced protein synthesis and muscle anabolism observed in COPD and in other chronic diseases could also be the consequence of the decrease in IGF-1 plasma concentrations, itself related to reduced GH secretion [11, 42, 59, 60]. In patients with low plasma testosterone, androgen therapy was associated with a decrease in subcutaneous fat and an increase in muscle mass [53, 57, 58, 61, 62], as well as an improvement of muscle force and functional capacities [53, 55, 57, 58, 61–66]. The rationale for androgen therapy in COPD patients is based on its ability to improve muscle mass and function [67], as well as nutritional status and clinical outcome.

## 4. Androgen Therapy: Clinical Results in COPD Patients 

In COPD patients, androgen therapy has been tested alone or together with physical exercise or nutritional supplementation.

### 4.1. Androgen Therapy Alone

Two studies have evaluated the effect of testosterone alone, that is, not integrated in a rehabilitation program, in COPD patients [68, 69] (Table 1). Oral testosterone analogue oxandrolone was given in a group of 128 outpatients (57 men and 71 women) with GOLD II-III COPD (forced expiratory volume (FEV) < 50%). A significant increase in fat-free mass was observed after four months of treatment [68]. However the 6-minute walking distance and spirometric data remained unchanged [68]. No differences were observed between men and women concerning treatment efficiency. Six women developed androgenic side effects (alopecia, cliteromegaly, hirsutism, and deepening of the voice) causing treatment discontinuation [68]. Nevertheless, testosterone supplementation was rather well tolerated in men as well as in women. In another group of 29 male outpatients with moderate to severe COPD (FEV < 60%), intramuscular testosterone was administered for 26 weeks with positive effects on body composition: fat-free mass increased by 1.1 kg, and fat mass decreased by 1.5% as compared to baseline. No effect was observed on lung function-associated parameters, such as blood gas analyses, spirometric data, 6-minute walking distance, and nocturnal oxygen saturation [69]. However an improvement in erectile function and sexual quality of life was reported. These two studies indicate that androgen therapy is able to improve muscle mass in COPD patients but, alone, is insufficient to improve lung function-associated parameters.

### 4.2. Androgen Therapy Combined with Physical Exercise

In two other studies, androgen therapy was combined with physical exercise [25, 71] (Table 1). A one-time 250 mg intramuscular injection of testosterone at baseline, followed by daily intake of 12 mg of oral stanozolol, was administered to ten undernourished (BMI < 20 kg/m^2^) male outpatients with GOLD II-III COPD [71]. The control group consisted of seven patients with similar characteristics who received placebo. All patients were part of an outpatient pulmonary rehabilitation program consisting in inspiratory muscle exercises (weeks 9 to 27) and cycle ergometer exercises (weeks 18 to 27). An increase in body weight was observed in the patients receiving anabolic steroids. On the contrary, in patients receiving placebo, body weight decreased and body mass index was significantly lower at the end of the study than in the intervention group. Fat-free mass (thigh circumference, arm muscle circumference, and dual energy X-ray absorptiometry) was significantly higher in the intervention group. No differences were found in maximal inspiratory pressure or 6-minute walking distance [71]. Casaburi et al. [25] studied the effects of a 10-week intramuscular testosterone administration (100 mg of testosterone enanthate per week) with or without resistance training (45 minutes three times per week) in a group of 47 men with moderate to severe (GOLD II to IV) COPD (mean FEV < 60% predicted) and low testosterone plasma concentrations (<400 ng/dL). The patients were divided in four groups: placebo without training; testosterone without training; placebo and training; testosterone and training. Only patients treated with testosterone (with or without training) reported a significant increase in fat-free mass. The group “testosterone and training” had the highest increase in muscle mass and force. This was also the only group in which significant increases in peak oxygen uptake, peak work rate, and lactic acidosis threshold were observed. A significant and similar increase in muscle force was observed in the groups “placebo and training” and “testosterone without training” [25]. These two studies indicate that the combination of testosterone and training is superior to testosterone or training alone to improve muscle mass and force.

### 4.3. Androgen Therapy (Testosterone) Combined with Physical Exercise and Nutritional Supplementation: Pulmonary Rehabilitation

Multimodal interventions including androgen therapy, a nutritional intervention, and an exercise program have also been assessed [70, 72] (Table 1). A double blind randomized trial in 217 patients with moderate to severe (GOLD II-III) COPD studied the effects of an 8-week nutritional intervention alone or combined with nandrolone decanoate IM injections every two weeks (50 mg for men and 25 mg for women) [70]. The nutritional intervention consisted in daily adding to the regular meals one oral nutritional supplement (ONS) which was a high caloric drink (200 mL = 420 kcal) administered in the early evening between 7:00 and 9:00 p.m. Nutritional intervention lasted at least until day 57. Patients were all admitted to an intensive inpatient pulmonary rehabilitation program. Both groups (nutrition alone and nutrition + testosterone) presented with a similar weight gain. Nevertheless, those treated with the testosterone analogue had a significantly higher increase of fat-free mass than the placebo group. Maximal inspiratory pressure, as a measure of respiratory muscle function, significantly increased after 8 weeks only in the group treated with nandrolone [70]. No differences concerning outcomes or side effects were observed between males and females [70]. A multimodal intervention was applied in a clinical trial in a group of moderate to severe COPD outpatients consisting in endurance physical exercises combined with oral nutritional supplements and oral testosterone undecanoate administration (80 mg or 40 mg twice daily for men and women, resp.) [72]. Nutritional intervention consisted ingiving three 120 mL ONS per day containing 180 kcal each. This triple intervention was applied for 90 days in 60 patients with chronic respiratory failure on long-term oxygen therapy and/or noninvasive ventilation. The control group consisted of 62 patients. Patients were undernourished (body mass index < 21 kg/m^2^ and low fat-free mass) (Table 1). In the “intervention group,” positive effects were reported on muscle mass, peak workload, quadriceps isometric force, and endurance time. No effect was observed on the 6-minute walking distance. Quality of life was improved only in women, independently of respiratory status, nutritional status, or compliance to therapy. Given these results, a stronger effect of hormonal therapy in women was speculated by the authors. One year after the end of the intervention (450 days of follow-up), survival was improved in the patients compliant to the pulmonary rehabilitation. Compliance was defined as having received at least 30% of two of the three treatments (exercise, ONS, and oral testosterone undecanoate) during the 3-month intervention [72]. These two trials clearly indicate the benefits of multimodal pulmonary rehabilitation associating physical exercise, oral nutritional supplementation, and androgen therapy, on muscle mass and force and clinical outcome of COPD patients.

### 4.4. Pulmonary Rehabilitation: A Pivotal Therapy for COPD

As stated in recent recommendations [41, 73], pulmonary rehabilitation should now be considered as a pivotal treatment, that is, not optional, for COPD patients functionally limited by dyspnea. It has been quoted with a grade A of evidence to improve dyspnea, exercise capacity, and health status [74]. The minimal content of a pulmonary rehabilitation program is to favour the regular practice of physical activity. Physical activity is initiated through at least three-weekly supervised sessions. This has to be followed by encouragements to maintain physical activities five times a week for 30 minutes each time. This training should contain progressive muscle resistance and aerobic endurance exercises to ensure muscle strength and endurance benefits [75, 76]. For more severely disabled patients, neuromuscular electrical stimulation could be helpful to improve muscle function [77]. Multimodal rehabilitation could be also beneficial in other chronic diseases. Specific care programs enclosing exercise training could delay the worsening of muscle atrophy in CHF patients [78, 79]. In patients with lung transplantation for respiratory chronic failure, renal failures, and type 2 diabetes, nutritional rehabilitation is associated with an improvement in the muscular oxidative metabolism [80–82] and a better survival [72]. The clinical trial NUTRICARD (ClinicalTrial.gov NCT01864733) is currently conducted to demonstrate whether nutritional rehabilitation would improve the clinical outcome of patients with CHF.

### 4.5. Safety of Androgen Therapy in COPD Patients

In the trials conducted in COPD patients and described above, androgen therapy has a safety profile. However, in these studies, androgen therapy was given during a short period of time while testosterone adverse effects appear mostly with longer treatment durations. The safety of androgen therapy has to be assessed on the long term. Extrapolating data from hypogonadic patients would be hazardous. Indeed, a significative proportion of COPD patients included in the different trials had no hypogonadism; in these patients, testosterone was used as a drug more than a hormone replacement therapy. Anyhow, physicians prescribing testosterone have to be aware of its potential side effects: cardio-vascular, sleep apnea syndrome worsening, prostate cancer, and polycythemia. Androgen therapy has to be specifically monitored [84]. Contraindications to androgen therapy are known (Table 2). Patients should also be informed about adverse effects such as mastodynia, gynecomastia, acne, and infertility. The risk for polycythemia seems particularly high in patients with chronic respiratory failure [84].

## 5. Perspectives

Three-month pulmonary rehabilitation is associated with an improved survival one year after the end of therapy [72]. However, the questions of the efficacy and the safety of a long-term pulmonary rehabilitation remain. Longer studies are warranted to better judge the safety profile of testosterone treatment in moderate to severe COPD patients. The success of pulmonary rehabilitation is the compliance of the patients to therapy. After the end of 3-month rehabilitation, patients are strongly encouraged to go on physical activity at home. This is difficult in daily practice since patients could lack willing and encouragement. This point is crucial and deserves improving the number of caregivers specialized in the area of pulmonary rehabilitation: pneumonologists, physiotherapists, nurses, ergotherapists,…… Research is clearly warranted to evaluate the long term effect of pulmonary rehabilitation and to determine the adapted frequency of long term physical exercise. Another important area of future research is the identification of clinical, molecular (myostatin, mitochondrial enzymes (cyclooxygenases, citrate synthase, ATPase,……), and transcriptional factors involved in protein metabolism (mTOR, Akt,……)) or related genetic factors involved in the clinical response to pulmonary rehabilitation. Indeed, identifying the patients who will have the best clinical benefits of pulmonary rehabilitation or androgen therapy is a key challenge.

## 6. Conclusion 

Fat-free mass loss and physical inactivity are key features of COPD and are related to impaired muscular oxidative metabolism. The preservation of muscle mass and function, through the protection of the mitochondrial oxidative metabolism, is an important challenge in the management of COPD patients. Androgen therapy improves muscle mass and force but is insufficient, alone, to improve lung function and clinical outcome. Nevertheless, in undernourished COPD patients, in combination with physical training and nutritional supplementations, androgen therapy is associated with increased fat-free mass and muscle force, together with increased peak workload and endurance time, and improved survival at least one year after the end of therapy. Pulmonary rehabilitation, associating physical exercise and nutritional supplements containing omega-3 polyunsaturated fatty acids, during at least three months, is the reference therapy of undernourished COPD patients. A short-term androgen therapy (3 months) could optimize the effects of rehabilitation in selected patients. Longer studies are warranted in order to identify whether mid- or long-term pulmonary rehabilitation could further improve the clinical outcome.

## Figures and Tables

**Table 1:** Clinical studies and trials having tested different protocols of androgen therapy in COPD patients.

Study	Drug/administration route	*N*	GOLD stage	Fat-free mass (FFM) loss	Method of FFM assessment	Dose	Frequency	Duration
Schols et al. [70]	Dandrolone decanoate/IM	217	II, III	FFM < 67% (men)/<63% (women) of ideal weight	BIA	M: 50 mg W: 25 mg	1x/2 weeks	6 weeks
Ferreira et al. [71]	Mixture of testosterone phenylpropionate, isocaproate, propionate, caproate/IM unique dose, and then stanozolol/PO	Total = 23, 17 completed study	II, III	—	DEXA	M: 250 mg M: 12 mg	“Charging dose” 1x/day	Unique dose 27 weeks
Yeh et al. [68]	Oxandrolone/PO	Total = 128 55 included in 4-month analysis	II, III	—	BIA	M: 10 mg W: 10 mg	2x/day 2x/day	16 weeks
Casaburi et al. [25]	Testosterone enanthate/IM	Total = 53 47 completed protocol	II, III, IV	—	DEXA	M: 100 mg	1x/week	10 weeks
Pison et al. [72]	Testosterone undecanoate/ PO	126	II, III, IV	<25th percentile of predicted FFMI: <18 (men) <15 (women)	BIA	M: 80 mg W: 40 mg	2x/day 2x/day	12 weeks
Svartberg et al. [69]	Testosterone enanthate/IM	29	II, III	—	DEXA	M: 250 mg	1x/4 weeks	26 weeks

BIA: bioelectrical impedance analysis; DEXA: dual energy X-ray absorptiometry; FFM: fat-free mass; FFMI: fat-free mass index (=FFM (kg)/height (m)^2^; IM: intramuscular; M: men; PO: per os; W: women.

**Table 2:** Current contraindications to androgen therapy in COPD patients (from [83]).

Expected survival < 6 months	
Previous history or actual hormono-dependent cancer (prostate or breast cancer)	
Prostatic nodule without any urological evaluation	
International prostate symptom score (IPSS) > 19/35	
Prostate-specific antigen (PSA) > 4 ng/mL (>3 ng/mL if familial (1st degree) history of prostate cancer or if black people)	
Previous history of psychotic disorders	
Acute heart failure	
Coronary heart disease in the past 6 months	
Sleep apnea in the absence of ventilator support	
Hematocrit > 50%	
ASAT or ALAT > 3 times of normal values	
Pulmonary artery hypertension	
Neuromuscular diseases	

ALAT: alanine aminotransferase; ASAT: aspartate aminotransferase.
